# Selenium Status Is Associated With Insulin Resistance Markers in Adults: Findings From the 2013 to 2018 National Health and Nutrition Examination Survey (NHANES)

**DOI:** 10.3389/fnut.2021.696024

**Published:** 2021-06-28

**Authors:** Barbara R. Cardoso, Sabine Braat, Ross M. Graham

**Affiliations:** ^1^Department of Nutrition, Dietetics and Food, Monash University, Melbourne, VIC, Australia; ^2^Departments of Medicine and Infectious Diseases at the Doherty Institute, University of Melbourne, Melbourne, VIC, Australia; ^3^Centre for Epidemiology and Biostatistics, Melbourne School of Population and Global Health, The University of Melbourne, Melbourne, VIC, Australia; ^4^Curtin Medical School, Curtin Health Innovation Research Institute, Curtin University, Bentley, WA, Australia

**Keywords:** selenium, diabetes, glucose, insulin, insulin resistance

## Abstract

Although literature has been consistently showing an increased risk of type 2 diabetes (T2DM) in populations with high exposure to selenium, there is a lack of information quantifying the association between diabetes-related markers and the nutritional status of selenium. Therefore, we aimed to investigate the association between blood selenium concentration and glucose markers in a representative sample of the US population, which is known to have moderate to high exposure to selenium. This cross-sectional analysis included 4,339 participants ≥18 years from the 2013 to 2018 National Health and Nutrition Examination Survey (NHANES). All participants were assessed for whole blood selenium concentration, fasting plasma insulin and glucose, HbA1c, and HOMA-IR (Homeostatic Model Assessment for Insulin Resistance). In this cohort, all participants presented with adequate selenium status [196.2 (SD: 0.9) μg/L] and 867 (15%) had diabetes mellitus. Selenium was positively associated with insulin, glucose and HOMA-IR in models adjusted for age and sex. When the models were further adjusted for smoking status, physical activity, metabolic syndrome and BMI, the associations with insulin and HOMA-IR remained but the association with glucose was no longer significant. A 10 μg/L increase in selenium was associated with 1.5% (95% CI: 0.4–2.6%) increase in insulin and 1.7% (95% CI: 0.5–2.9%) increase in HOMA-IR in fully adjusted models. There was no evidence of an association between selenium and diabetes prevalence. Our findings corroborate the notion that selenium supplementation should not be encouraged in populations with high dietary intake of selenium.

## Introduction

Selenium is an essential micronutrient to human life, as it is required for the synthesis of the 21st amino acid selenocysteine, the defining feature of 25 selenoproteins identified in humans. The selenoproteome is small and functionally diverse. Selenoproteins are recognized as crucial for antioxidant response, as well as for immune system and thyroid hormone regulation, and heavy metal and xenobiotic detoxification [reviewed by Roman et al. (1)], but around half the identified human selenoproteins remain largely uncharacterized (2).

Diet is the principal source of selenium, and selenium intake is reflected by corresponding concentrations in the soil where crops are grown (3). Inadequate selenium intake, which affects one in seven people in the world (3), has been associated with increased risk of cancer (4), neurodegenerative diseases (5–7) and thyroid dysfunction (8, 9). This has led to empirical health advice recommending increasing selenium intake through diet or supplementation. As a result, selenium supplements have been widely consumed under the understanding that “the more the better.” Nonetheless, recent studies have indicated that high selenium consumption is associated with an increased risk of chronic diseases such as diabetes (10) and non-alcoholic fatty liver disease (11), and all-cause mortality (12). These studies corroborate the hypothesis that the metabolic outcomes of selenium in the human body follow a U-shaped curve (13, 14), meaning that selenium intake within the correct range is critical for human well-being, with either too high or too low being prejudicial.

First evidence linking high selenium consumption and type 2 diabetes (T2DM) was derived from a secondary analysis of a trial where selenium (200 μg/day) was provided to over 1,200 non-melanoma skin cancer patients aiming to evaluate the efficacy of selenium supplementation for prevention of cancer. After 7.7 years of intervention, no cancer protective effects were observed and, as an unexpected secondary outcome of the trial, the researchers reported an increased risk for T2DM in the selenium group compared to placebo (hazard ratio: 1.55; 95% CI: 1.03, 2.33) (15). More recently, meta-analyses of human studies revealed that plasma selenium concentrations of 140 μg/L were associated with a 3.6-fold increased risk of T2DM when compared with the reference category (45 μg/L) (10), although data from randomized clinical trials (RCTs) reported by a systematic review did not confirm an eventual negative effect of selenium on the incidence of the disease (Odds Ratio: 1.18; 95% CI: 0.95, 1.47) (16). Despite limited information on selenium supplementation to individuals with T2DM, a systematic review looking into the effectiveness of selenium supplementation in adults with T2DM reported that selenium treatment had no effect on HbA1c, and fasting blood glucose and insulin in four RCTs. However, one study reported an increase in fasting plasma glucose, and two studies reported a decrease in insulin resistance, assessed as fasting plasma insulin, HOMA-IR (Homeostatic Model Assessment for Insulin Resistance) and HOMA-B (Homeostasis Model Assessment of β-cell dysfunction) (17).

Although literature has been consistently showing an increased risk of T2DM in populations with high exposure to selenium, as recently demonstrated in Americans assessed in the National Health and Nutrition Examination Survey (NHANES) (18), there is a lack of information identifying diabetes-related markers associated with nutritional status of selenium. Therefore, we aimed to investigate the association between selenium status, measured as blood selenium, and glucose markers in a representative sample of the US population, which is known to have moderate to high exposure to selenium (19, 20).

## Methods

### Study Population

This cross-sectional analysis included participants ≥18 years of age in three National Health and Nutrition Examination Survey (NHANES) cycles: 2013–14, 2015–16, and 2017–18. NHANES was conducted by both the Center for Disease Control and the National Center for Health Statistics, and utilizes a complex, multistage, probability-sampling procedure to provide nationally representative estimates on the health and nutritional status of non-institutionalized US residents (21). The NHANES protocol was approved by the National Center for Health Statistics (NCHS) Research Ethics Review Board. Informed consent was obtained from all participants included in the study (22).

**Figure 1:**
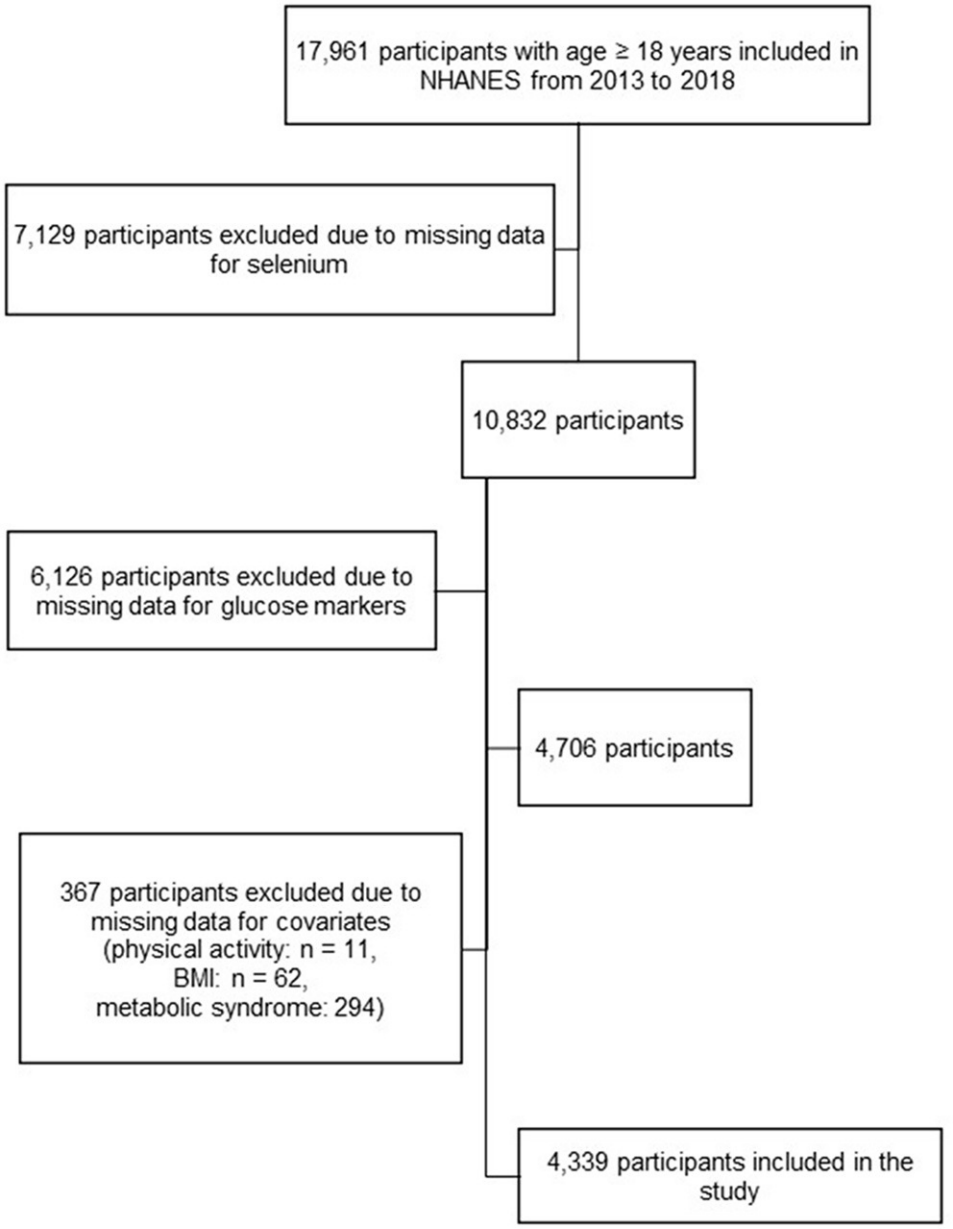
Selection of the study population, from the National Health and Nutrition Examination Survey (NHANES) from 2013 to 2018.

In the NHANES cycles included in this study, a total of 17,961 participants ≥ 18 years old were interviewed. According to the complex NHANES survey design, only one-half sample from participants in the NHANES aged 12 years and older was randomly eligible for blood selenium assessment; those who were examined in the morning session were eligible for fasting glucose and insulin assessment. Individuals were excluded from our analysis if they had missing data for: (i) selenium; (ii) glucose or insulin markers; (iii) any variable necessary for the definition of metabolic syndrome (blood pressure, triglycerides, HDL, waist circumference, blood glucose, with parameters as defined under the Covariates heading, below); (iv) covariates included in the models (physical activity, BMI). After exclusions, the total number of participants for our analysis was 4,339 (Figure 1).

### Glucose and Insulin Measures

Glycohemoglobin (Hb1Ac) was measured using a Tosoh Automated Glycohemoglobin Analyser HLC-723G8. Fasting glucose was measured in the serum enzymatically by a hexokinase-mediated reaction using a chemistry analyser (Roche/Hitachi Cocas C Chemistry Analyser). Serum insulin was measured by the Elecsys 2010 Insulin chemiluminescent sandwich immunoassay. HOMA-IR was calculated according to the formula: fasting insulin (μU/L) × fasting glucose (mmol/L)/22.5. Individuals were considered as having diabetes if they reported current use of prescribed medication (including insulin) to control glucose levels, or presented with HbA1c ≥ 6.5% or fasting glucose ≥ 126 mg/dL (23).

### Selenium

Selenium was measured in whole blood by inductively coupled plasma mass spectrometry (ICP-MS), that monitored the ion intensity at m/z 80 (^80^Se). Polyatomic interferences in the analysis were reduced by using methane as a reaction gas. The lower limit of detection was 24.5 μg/L; no values below this were reported in the dataset (24).

### Covariates

Participants completed a self-reported demographic questionnaire that included questions about age, sex, race, and smoking status. Racial groups were categorized as Mexican American, other Hispanic, Non-Hispanic White, Non-Hispanic Black, Non-Hispanic Asian, Other non-Hispanic. Current smokers were identified as those who responded “every day” or “some days” to the question “Do you now smoke cigarettes?.” Anthropometric measurements were performed by trained health technicians while the participants wore a standard examination gown. Waist circumference was measured at the superior lateral border of participants' iliac crest and recorded as cm. A digital scale was used to measure body weight, and height was measured using a stadiometer. BMI was calculated as weight (kg) divided by squared height (m^2^), and then rounded to one decimal place. Physical activity was assessed using the Global Physical Activity Questionnaire. Sum of daily moderate and vigorous physical activity was calculated by multiplying the frequency per week by the duration (minutes) of the physical activity divided by seven. Z-scores for moderate/vigorous activity were generated using the mean and standard deviation of the NHANES ≥ 18 years population. Metabolic syndrome was defined according to the harmonized definition that considers the presence of at least three of the following conditions: (i) elevated waist circumference (≥88 cm for women or ≥102 cm for men; (ii) elevated triglyceride levels (≥150 mg/dL); (iii) low HDL levels, characterized as ≤50 mg/dL for women, ≤40 mg/dL for men, or current use of prescribed medication to treat high cholesterol; (iv) elevated blood pressure, identified as systolic blood pressure ≥130 mmHg, diastolic blood pressure ≥85 mmHg, or current use of prescribed medication to treat high blood pressure; (v) elevated fasting blood glucose levels, characterized by ≥100 mg/dL or current use of prescribed medication or insulin to treat hyperglycaemia (25).

### Statistical Analysis

Data analyses included complete data of survey participants and used sample weights provided by NHANES for blood selenium in order to account for the complex NHANES survey design including nonresponse and oversampling. Demographic and clinical characteristics were presented as mean with standard deviation (SD) for continuous variables, or number (% weighted) for categorical variables. The glucose markers were log (base e) transformed based on inspection of the quantile-quantile plots. The association between selenium concentration and the outcomes (log HbA1c, log glucose, log insulin, and log HOMA-IR) was examined using two multivariable linear regression models. Model 1 was adjusted for age and sex; Model 2 was further adjusted for smoking status (smokers/non-smokers), physical activity (*Z*-score), BMI (kg/m^2^), and metabolic syndrome (yes/no). The linearity of the associations was explored visually by fitting a restricted cubic spline with four knots (at 5th, 35th, 65th, and 95th percentiles of selenium concentration). The association between the prevalence of diabetes and selenium concentration was investigated using Poisson regression using selenium as a continuous variable. Selenium concentration was compared between individuals with and without diabetes using linear regression. Two models with the same covariates as mentioned above for models 1 and 2 were tested.

Effect modification analyses were performed in order to explore whether the relationship between selenium and the outcomes was modified according to age, sex, presence of metabolic syndrome or diabetes, smoking status, BMI, or physical activity by including the interaction term between selenium and one of the covariates in model 2.

Statistical analysis was performed with STATA/SE 16.0 for Windows (StataCorp LLC). Confidence intervals and *P-*values were reported two-sided without adjustment for multiple testing.

## Results

Table 1 displays the characteristics of NHANES participants included in this analysis. The average age of the participants was 47.3 years. Females accounted for 51% of the study sample, and the majority self-reported as non-Hispanic white (64%). The study population presented with similar demographic characteristics when compared with the excluded individuals (Supplementary Table 1). Overall, BMI was in the overweight range, with 1,698 (39%) individuals presenting with obesity (BMI ≥ 30). HDL-cholesterol was below reference cut-offs (≥60 mg/dL), while blood pressure was considered within normal ranges overall. Prevalence of metabolic syndrome in this population was 43% and 15% of the participants met the criteria for diabetes mellitus.

**Table 1:** Demographic and clinical characteristics of US adults in the NHANES 2013–2018.

	**Study sample (*n* = 4,339)**
Age, years	47.3 (0.4)
Females, *n* (%)	2,242 (50.9%)
**Racial group**, ***n*** **(%)**	
*Mexican American*	662 (9.4%)
*Other Hispanic*	474 (6.5%)
*Non-Hispanic White*	1,581 (64.4%)
*Non-Hispanic Black*	891 (10.4 %)
*Non-Hispanic Asian*	549 (5.3%)
*Other non-Hispanic*	182 (4.0%)
**Smoking status**, ***n*** **(%)**	
*Current smoker*	780 (16.8%)
*Non-smoker*	3,559 (83.2%)
Physical activity, *z*-score	−0.03 (0.02)
BMI, kg/m^2^	29.3 (0.2)
Waist circumference, cm	100.1 (0.5)
HbA1c, %	5.64 (0.02)
Fasting insulin, U/mL	12.5 (0.3)
Fasting glucose, mg/dL	6.00 (0.03)
HOMA-IR	3.67 (0.10)
HDL-cholesterol, mg/dL	55.0 (0.5)
**Blood pressure, mm/Hg^a^**	
*Systolic*	122.4 (0.4)
*Diastolic*	70.0 (0.4)
Triglycerides, mg/dL	126.0 (1.9)
Metabolic syndrome, *n* (%)	2,012 (42.9%)
Diabetes mellitus, *n* (%)	867 (15.0%)
Selenium, μg/L	196.2 (0.9)

*All values are mean with standard deviation (SD) or number (proportions, %)*.

a *n = 4,287; Metabolic syndrome was defined according to Alberti et al. (25); Diabetes mellitus was defined based on participants' self-reported current use of prescribed medication (including insulin) to control glucose levels, or HbA1c ≥ 6.5% or fasting glucose ≥ 126 mg/dL (23). BMI, Body Mass Index*.

All the participants were considered selenium-replete given that selenium in blood was above the cut-off established reflecting the minimum concentration to maximize activity of the selenoprotein glutathione peroxidase (GPx; 84–100 μg/L) (26) (Table 1).

**Figure 2:**
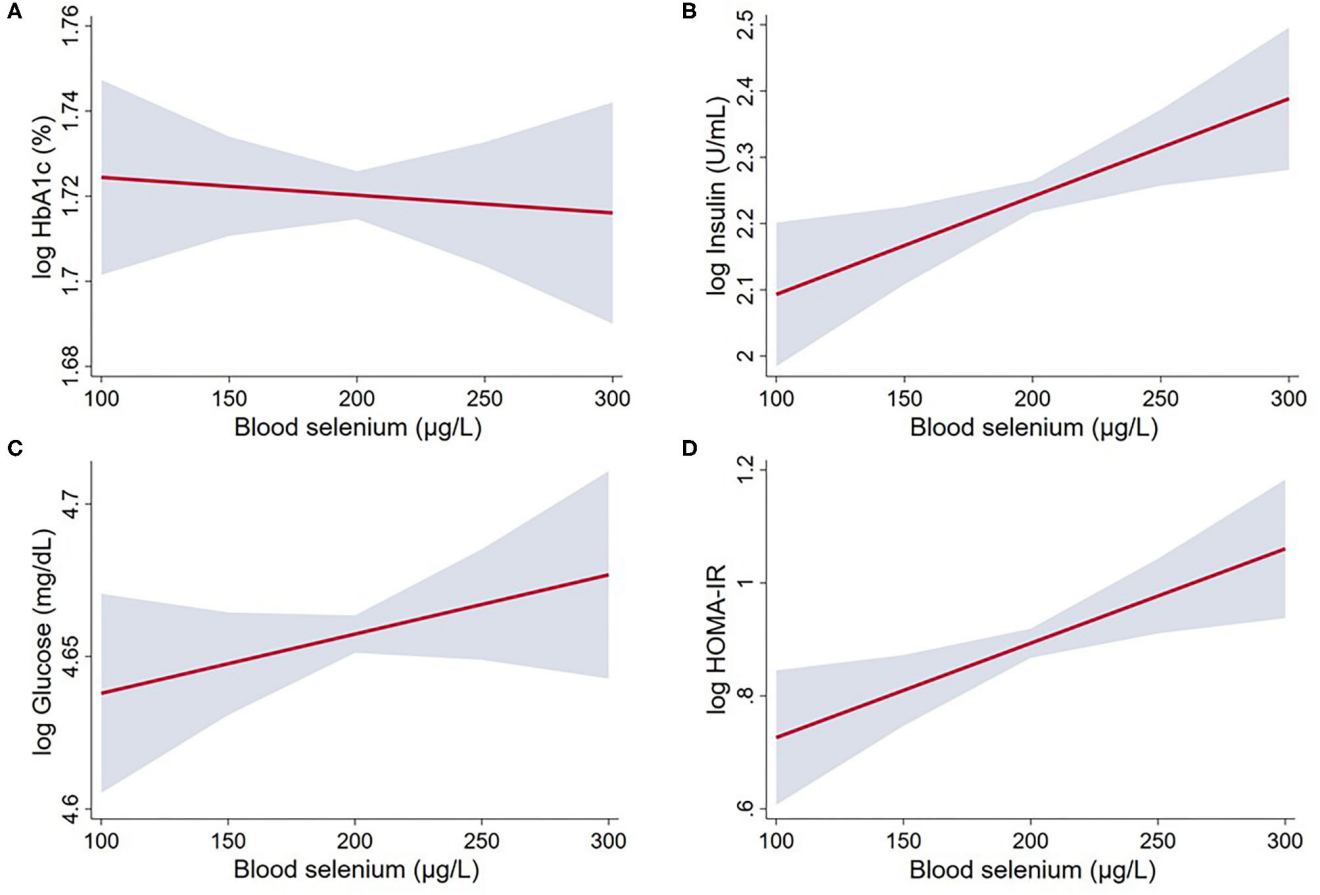
Linear regression between whole blood selenium concentration and glucose and insulin markers in US adults from NHANES 2013 to 2018: **(A)** log HbA1c; **(B)** log Insulin; **(C)** log Glucose; and **(D)** log HOMA-IR. The associations were adjusted for age, sex, smoking status (smoker/non-smoker), physical activity, metabolic syndrome, BMI (Model 2). Gray area represents the 95% confidence intervals. Statistical parameters for these variables are given in Table 2.

In the model adjusted for sex and age, selenium concentration was positively and linearly associated with insulin, fasting glucose and HOMA-IR. After further adjustment for smoking status, physical activity and presence of metabolic syndrome, a 10 μg/L increase in selenium was associated with 1.5% (95% CI: 0.4–2.6%) increase in insulin and 1.7% (95% CI: 0.5–2.9%) increase in HOMA-IR (Table 2; Figure 2). Selenium concentration was not significantly associated with the prevalence of diabetes mellitus in this population in either of the two models (Model 1: Estimate: 1.00, 95% CI: 1.00, 1.01; Model 2: Estimate: 1.00, 95% CI: 1.00, 1.00). No significant difference was observed in selenium concentration between individuals with and without diabetes (mean difference: −3.9 μg/L, 95% CI: −8.8 to 0.85, *P* = 0.104).

**Table 2:** Associations between whole blood selenium concentration and glucose and insulin markers in US adults from NHANES 2013 to 2018.

	**Model 1^a^^,^^b^**		**Model 2^a^^,^^c^**	
	**Estimate (95% CI)**	***P*-value**	**Estimate (95% CI)**	***P*-value**
HbA1c, %	1.0002 (−1.002, 1.0023)	0.825	−1.0004 (−1.003, 1.002)	0.726
Insulin, U/mL	1.020 (1.007, 1.030)	0.003^*^	1.015 (1.004, 1.026)	0.007^*^
Glucose, mg/dL	1.003 (1.00, 1.007)	0.037^*^	1.002 (−1.001, 1.005)	0.239
HOMA-IR	1.024 (1.009, 1.039)	0.002^*^	1.017 (1.005, 1.029)	0.007^*^

a *The models were fitted using log-transformed outcomes and are presented on the original scale for 10 μg/L increase in selenium;*

b *Model 1 adjusted for age and sex;*

c *Model 2 adjusted for age, sex, smoking status (smoker/non-smoker), physical activity, metabolic syndrome, and BMI*.

* *P < 0.05*.

Seven models for sensitive analysis were performed containing interaction terms between selenium and one of the covariates included in the fully adjusted model, but no interaction was identified (all *P* ≥ 0.2) (Supplementary Table 2).

## Discussion

Despite current evidence showing an increased risk of diabetes amongst individuals with higher selenium intake and increased concentration in different blood markers, the literature lacks information on the association between selenium status and diabetes-related markers. By investigating diabetes-related markers, our study is designed to shed light on potential mechanisms disturbed by high levels of circulating selenium. In this cross-sectional analysis of a selenium-replete population of US adults, blood selenium concentration was positively associated with insulin concentration and HOMA-IR, markers of insulin resistance. Selenium status was also associated with fasting glucose; however this association was no longer significant when the model was further adjusted for smoking status, physical activity, metabolic syndrome and BMI. Finally, there was no evidence of an association between selenium and diabetes mellitus.

To our knowledge, our study is the first to investigate the association between selenium status and diabetes-related markers. In our analysis, only markers of insulin resistance (insulin concentration and HOMA-IR), but not glucose, were associated with selenium in the fully adjusted model. Our findings corroborate the hypothesis that high selenium status disturbs insulin metabolism leading to hyperinsulinemia (27, 28), but the effect on glucose is not as strong, as it can be mitigated when controlling strong risk factors for T2DM such as body weight and metabolic syndrome. In our study, no association between selenium status and the prevalence of diabetes was observed, which counteracts the findings reported in two other studies conducted in the NHANES population (18, 29). Nonetheless, it is important to mention some key differences between our analysis and those studies. Bleys et al. (29) reported that the highest quintile of selenium in plasma (≥137.66 μg/L) was associated with OR = 1.57 (95% CI: 1.16, 2.13) when compared with the lowest quintile (<111.62 μg/L) in US adults, although no trend was observed for quintiles 2–4. In that study, selenium was assessed in plasma, which represents a short-term marker and therefore is more sensitive to acute dietary changes. Furthermore, the definition of diabetes used in that study was a self-report of a physician diagnosis of diabetes, which may be biased and result in prevalence of diabetes being over-estimated. Our study used more reliable criteria to define diabetes, reducing the risk of bias. More recently, Moon et al. (18) revealed that the increase of 10 μg/L in blood selenium increased the prevalence of diabetes by 12% (OR: 1.12; 95% CI: 1.06–1.18) in adults from the 2011 to 2014 NHANES. Nonetheless, unlike our study, Moon et al. did not consider Hb1Ac in the diagnosis of diabetes; furthermore, they did not consider smoking status or physical activity as covariates, although these are two important risk factors for diabetes. Additionally, while we adjusted our model for metabolic syndrome, they adjusted their models for only some metabolic syndrome-related factors such as hypertension and dyslipidaemia.

Animal experiments have been conducted to elucidate the mechanisms involved in the association between selenium nutritional status and the risk of T2DM. These studies have associated the upregulation of the selenoproteins glutathione peroxidase 1 (GPX1), methionine sulfoxide reductase B1 (MSRB1), selenoprotein S (SELENOS) (27) and selenoprotein P (SELENOP) (30) induced by high selenium intake with hyperglycaemia, decreased insulin sensitivity and liver triglyceride concentrations. It was hypothesized that increased synthesis of these antioxidant selenoproteins diminishes intracellular reactive oxygen species derived from glucose metabolism and disturbs key regulators of pancreatic beta-cells, leading to chronic hyperinsulinaemia (27, 28). An experimental animal model of diabetes revealed that neutralization of SELENOP improved glucose tolerance and insulin secretion (31), suggesting that this selenoprotein may play a particularly important role in the pathogenesis of T2DM.

Taking a different perspective, Schomburg (32) hypothesized that rather than high selenium being a cause for insulin resistance, diabetes potentially causes increased synthesis of SELENOP by the liver, which leads to increased circulating selenium. Under this hypothesis, the primary cause of the association between selenium and diabetes is the presence of insulin resistance that raises the synthesis of SELENOP, the main selenium transporter. When developing this hypothesis, Schomburg (32) raised concerns around “young autoimmune-disease prone women who wished to supplement and correct their selenium deficiency” but are precluded from doing so because they are afraid of developing diabetes due to the supplementation. Findings from a systematic review that included RCTs on selenium supplementation to individuals with T2DM revealed inconsistent effects of selenium on the main glucose and insulin markers (17). While no effect of selenium was seen in two out of the four included studies, insulin resistance markers (plasma insulin, HOMA-IR and HOMA-B) were decreased after the intervention in comparison to the placebo group in two studies. Nonetheless, no selenium status was assessed in these studies, and therefore it is not possible to establish if the positive effects were due to a recovery from selenium deficient nutritional status.

High selenium intake has been associated with increased risk of diabetes in observational and controlled trial studies. A meta-analysis conducted with four studies which assessed the association between dietary selenium and risk of diabetes revealed that, in comparison with a selenium intake of 23 μg/day, an intake of 50 μg/day was associated with a relative risk (RR) of 1.5 (95% CI: 1.1, 1.9), and an intake of 75 μg/day was associated with an even higher RR (RR: 1.9; 95% CI: 1.4, 2.7) (10). We here emphasize the U-shaped metabolic effects of selenium, where both too little or too much selenium is detrimental to human health (4, 10, 14). We found that the population assessed in this analysis is selenium-replete, in accordance with other studies within the NHANES that demonstrated a high consumption via diet and supplementation (19, 20). Thus, a linear relationship, representing the right-hand arm of a U-shaped association, was observed. Although we cannot extrapolate our findings to selenium-deficient populations, through the lens of a U-shaped dose-response, it is possible that a selenium-deficient population would in fact benefit from supplementation. Further analysis to determine potential benefits of selenium supplementation in selenium-deficient individuals is required.

Our analysis encompassed the examination of sex differences in the association between selenium status and glucose markers, but no significant sex interaction was observed. This analysis is critical due to previously reported sex differences in selenium biology (33, 34), and should be included in every study.

A key strength of this study relies on the survey and analytical methods, as well as the controlled protocols used by the NHANES. Furthermore, we used blood selenium as a marker, which is the preferred option to assess selenium status in populations with high exposure to selenium as it does not plateau like plasma and is very responsive to selenium intake (35). A limitation of this study is associated with the cross-sectional nature of the study design, which precludes inference of causation.

## Conclusions

In this analysis that included a representative sample of US adults with selenium-replete nutritional status, selenium was positively associated with markers of insulin resistance, such as plasma insulin concentration and HOMA-IR, independently of other risk factors for T2DM such as smoking status, physical activity, metabolic syndrome and BMI. Our findings corroborate the notion that selenium supplementation should not be encouraged in populations with high dietary intake of selenium.

## Data Availability Statement

Publicly available datasets were analyzed in this study. This data can be found here: https://wwwn.cdc.gov/nchs/nhanes/.

## Author Contributions

BRC: conceptualization, data curation, data analysis, and writing (original and final draft). SB: data analysis and writing (review and editing). RMG: writing (review and editing). All authors contributed to the article and approved the submitted version.

## Conflict of Interest

The authors declare that the research was conducted in the absence of any commercial or financial relationships that could be construed as a potential conflict of interest.
