# Supplementary material for: Selenium Status Is Associated With Insulin Resistance Markers in Adults: Findings From the 2013 to 2018 National Health and Nutrition Examination Survey (NHANES)
Source: Front Nutr. 2021 Jun 28;8:696024. doi: 10.3389/fnut.2021.696024 (PMC8273176; doi:10.3389/fnut.2021.696024)
Supplement: Supplementary file 1 [file Table_1.DOCX]

Supplementary Material

# Supplementary Tables

**Table S1.** Demographic characteristics of adults (≥ 18 years old) in the NHANES 2013–2018.

|  | **Study sample**  **(n=4,339)** | **Excluded sample**  **(n=13,622)** |
| --- | --- | --- |
| Age, years | 47.3 (0.4) | 46.9 (0.3) |
| Females, n (%) | 2242 (50.9%) | 7076 (52.1%) |
| Racial Group, n (%) |  |  |
| *Mexican American* | 662 (9.4%) | 2042 (9.1%) |
| *Other Hispanic* | 474 (6.5%) | 1407 (6.3%) |
| *Non-Hispanic White* | 1581 (64.4%) | 4936 (63.4%) |
| *Non-Hispanic Black* | 891 (10.4 %) | 2971 (11.8%) |
| *Non-Hispanic Asian* | 549 (5.3%) | 1732 (5.8%) |
| *Other non-Hispanic* | 182 (4.0%) | 534 (3.6%) |
| Smoking status, n (%) |  |  |
| *Current smoker* | 780 (16.8%) | 2573 (19.0%) |
| *Non-smoker* | 3559 (83.2%) | 11,036 (81.0%) |
| Physical activity, z-score | -0.03 (0.02) | -0.02 (0.01) |

All values are mean with standard deviation (SD) or number (proportions, %). ^1^ Comparisons between groups performed with Student t-test or Pearson chi-squared test.

**Table S2.** Effect modification analyses of the association between selenium and glucose and insulin markers in US adults from NHANES 2013–2018.

|  | **HbA1c, %** | | **Insulin, U/mL** | | **Glucose, mg/dL** | | | **HOMA-IR** | |
| --- | --- | --- | --- | --- | --- | --- | --- | --- | --- |
| **Models^1^** | **Estimate**  **(95% CI)** | ***P*-value** | **Estimate**  **(95% CI)** | ***P*-value** | **Estimate**  **(95% CI)** | ***P*-value** | | **Estimate**  **(95% CI)** | ***P*-value** |
| Age × Selenium | 1.000  (1.000, 1.000) | 0.367 | 1.000  (0.999, 1.000) | 0.267 | 1.000  (1.000, 1.000) | 0.227 | 1.000  (0.999, 1.000) | | 0.476 |
| Sex × Selenium | 1.002  (0.998, 1.006) | 0.295 | 0.998  (0.976, 1.024) | 0.983 | 0.999  (0.992, 1.005) | 0.707 | 0.998  (0.972, 1.026) | | 0.916 |
| Metabolic syndrome × Selenium | 0.999  (0.995, 1.003) | 0.777 | 0.985  (0.964, 1.007) | 0.189 | 0.999  (0.993, 1.005) | 0.764 | 0.985  (0.958, 1.010) | | 0.229 |
| Diabetes × Selenium | 1.002  (0.991, 1.014) | 0.611 | 1.013  (0.984, 1.042) | 0.378 | 1.002  (0.986, 1.017) | 0.808 | 1.015  (0.975, 1.056) | | 0.470 |
| Smoking status × Selenium | 0.999  (0.993, 1.004) | 0.660 | 1.000  (0.970, 1.031) | 0.992 | 0.996  (0.989, 1.003) | 0.225 | 0.995  (0.963, 1.029) | | 0.787 |
| BMI × Selenium | 1.000  (1.000, 1.000) | 0.869 | 0.999  (0.997, 1.001) | 0.504 | 1.000  (1.000, 1.000) | 0.195 | 0.999  (0.997, 1.002) | | 0.706 |
| Physical activity × Selenium | 1.000  (1.000, 1.000) | 0.921 | 1.000  (0.998, 1.000) | 0.773 | 1.000  (1.000, 1.000) | 0.913 | 1.000  (1.000, 1.000) | | 0.815 |

^1^ The models were fitted using log-transformed outcomes, they are presented on the original scale for 10 µg/L increase in selenium; Models were adjusted for age, sex, smoking status (smoker/non-smoker), physical activity, metabolic syndrome, BMI. For each model, selenium, the covariate and their interaction were included.
